# Social participation, subjective well-being, and cognitive function as serial mediators between tooth loss and functional limitations in older Chinese adults.

**DOI:** 10.1186/s12889-024-18255-w

**Published:** 2024-03-14

**Authors:** Weibo Ma, Pengchen Liang, Bei Wu, Ying Yu, Qiusi Shi, Renyao Zhong

**Affiliations:** 1 School of Public Administration, Faculty of Economics and Management, East China Normal University, 3663 Zhongshan Road, Putuo District, 200062 Shanghai, China; 2 School of Microelectronics, Shanghai University, Shanghai, China; 3 NYU Aging Incubator and Hartford Institute for Geriatric Nursing, New York, USA; 4 Shanghai University of Medicine and Health Sciences, Shanghai, China

**Keywords:** Tooth loss, Functional limitation, Social participation, Subjective well-being, Cognitive function, Serial mediation model

## Abstract

**Background:**

Although tooth loss appears to be related to functional limitations, the mechanisms that underpin this relationship are unknown. We sought to address this knowledge gap by examining a multiple mediation hypothesis whereby tooth loss is predicted to indirectly affect functional limitations through social participation, subjective well-being, and cognitive function.

**Methods:**

This study included 7,629 Chinese adults from the 2017/2018 Chinese Longitudinal Healthy Longevity Survey wave. The serial mediation effects were examined using Model 6 in the Hayes’ PROCESS macro for SPSS.

**Results:**

Tooth loss was significantly related to functional limitations. There was a direct (β = − 0.0308; 95% CI, − 0.0131 to − 0.0036) and indirect (β = − 0.0068; 95% CI, − 0.0096 to − 0.0041) association between tooth loss and instrumental activities of daily living (IADL) limitations, but only an indirect correlation with activities of daily living (ADL) limitations (β = − 0.0188; 95% CI, − 0.0259 to − 0.0121). Social participation, subjective well-being, and cognitive function serially mediated the relationship between tooth loss and ADL/IADL limitations.

**Conclusion:**

The association between tooth loss and functional limitations is serially mediated by social participation, subjective well-being, and cognitive function. Our findings underscore the necessity of considering psychological and social factors as integrated healthcare approaches for the functional health of older adults.

**Supplementary Information:**

The online version contains supplementary material available at 10.1186/s12889-024-18255-w.

## Introduction

China is ageing much faster than other low- and middle-income countries [1]. Facing the huge economic and social burden of aging, the country considers how to realize healthy aging as an important strategic measure. The key to healthy aging is maintaining functional ability despite having chronic conditions [2]. Forecasts suggest that by 2060, the number of older adults with functional limitations in China will increase to 23.9 million [3]. Therefore, it is imperative to fully understand the mechanisms associated with functional limitations and explore the intervening factors to prevent or delay their development.

As the population ages, the cumulative effect of oral health on healthy aging will become increasingly relevant. Many studies have confirmed that oral health is closely related to the risk of developing functional limitations [4]. Tooth loss, a key indicator of oral health, is no exception [5]. Studies have shown that tooth loss was linked to problems with instrumental activities of daily living (IADL), activities of daily living (ADL), and mobility [4–6]. Possible pathways underlying the associations between tooth loss and disability could include biological factors such as oral inflammation [7] and poor nutrition [8]; however, the role of psychological and social mechanisms remains unclear.

The influence of tooth loss on personal social behavior and psychology cannot be ignored. Tooth loss can have significant aesthetic consequences that might lead to social embarrassment or withdrawal. Cross-sectional and cohort studies have shown that tooth loss or having few teeth negatively impacts social participation [9–11]. Dietary and communication difficulties due to tooth loss reduce social participation and lead to frustration in self-esteem, thus reducing subjective well-being (SWB) [12–13]. A study in Japan reported an independent correlation between the number of teeth and SWB [14]. Another cross-sectional study found that older people with tooth loss were 65% more likely to have low SWB than those who did not report tooth loss [15]. Furthermore, tooth loss could cause cognitive impairment. A meta-analysis found that each additional tooth lost increased the relative risk of cognitive impairment by 0.014 [16]. Regardless of whether it is social participation, SWB, or cognitive function, tooth loss is an important measure of healthy aging realization and an important factor in maintaining a good functional state [2]. Low-quality social participation, poor SWB, and cognitive impairment could negatively affect the motivation for physical activity and self-care, resulting in functional limitations. Although the existing literature mainly focuses on the direct relationship between functional limitations and social participation, SWB, and cognitive function [16–19], the disablement process model emphasizes that psychological factors might be important intermediaries in the association between chronic diseases and functional limitations [20]. Therefore, this study hypothesized that social participation, SWB, and cognitive function mediate the relationship between tooth loss and functional limitations.

Activity theory, one of the most popular theories in the field of SWB, emphasizes the key roles social participation plays in increasing SWB [21]. Interestingly, social participation and SWB have protective effects on cognitive function [22–23] and the risk of disability or functional limitations [24–25]. Although cognitive impairment is closely related to functional limitations, theory and research have shown that poor cognitive impairment precedes functional deterioration [26–27]. Given the above theoretical and research support, social participation, SWB, and cognitive function might constitute a serial mediating path that impacts the association between tooth loss and functional limitations; however, this hypothesis needs further research and verification.

**Fig. 1:**
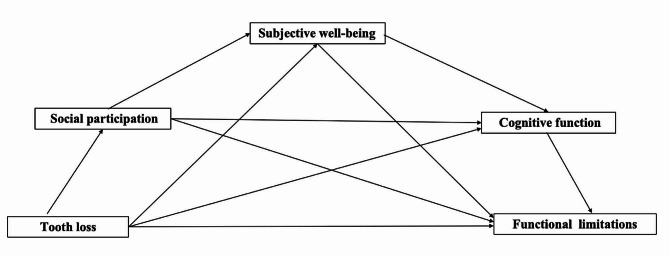
A hypothetical model of the study

This study had two primary aims. First, to examine the association between tooth loss and functional limitations. Second, to quantify the extent to which social participation, SWB, and cognitive function directly or serially mediate the association between tooth loss and functional limitations. Figure 1 illustrates the proposed research model and puts forward the following assumptions:

### Hypothesis 1

Tooth loss is positively associated with functional limitations.

### Hypothesis 2

Social participation mediates the relationship between tooth loss and functional limitations.

### Hypothesis 3

SWB mediates the relationship between tooth loss and functional limitations.

### Hypothesis 4

Cognitive function mediates the relationship between tooth loss and functional limitations.

### Hypothesis 5

Social participation, SWB, and cognitive function play a serial mediating role in the relationship between tooth loss and functional limitations.

## Methods

### Participants

**Fig. 2:**
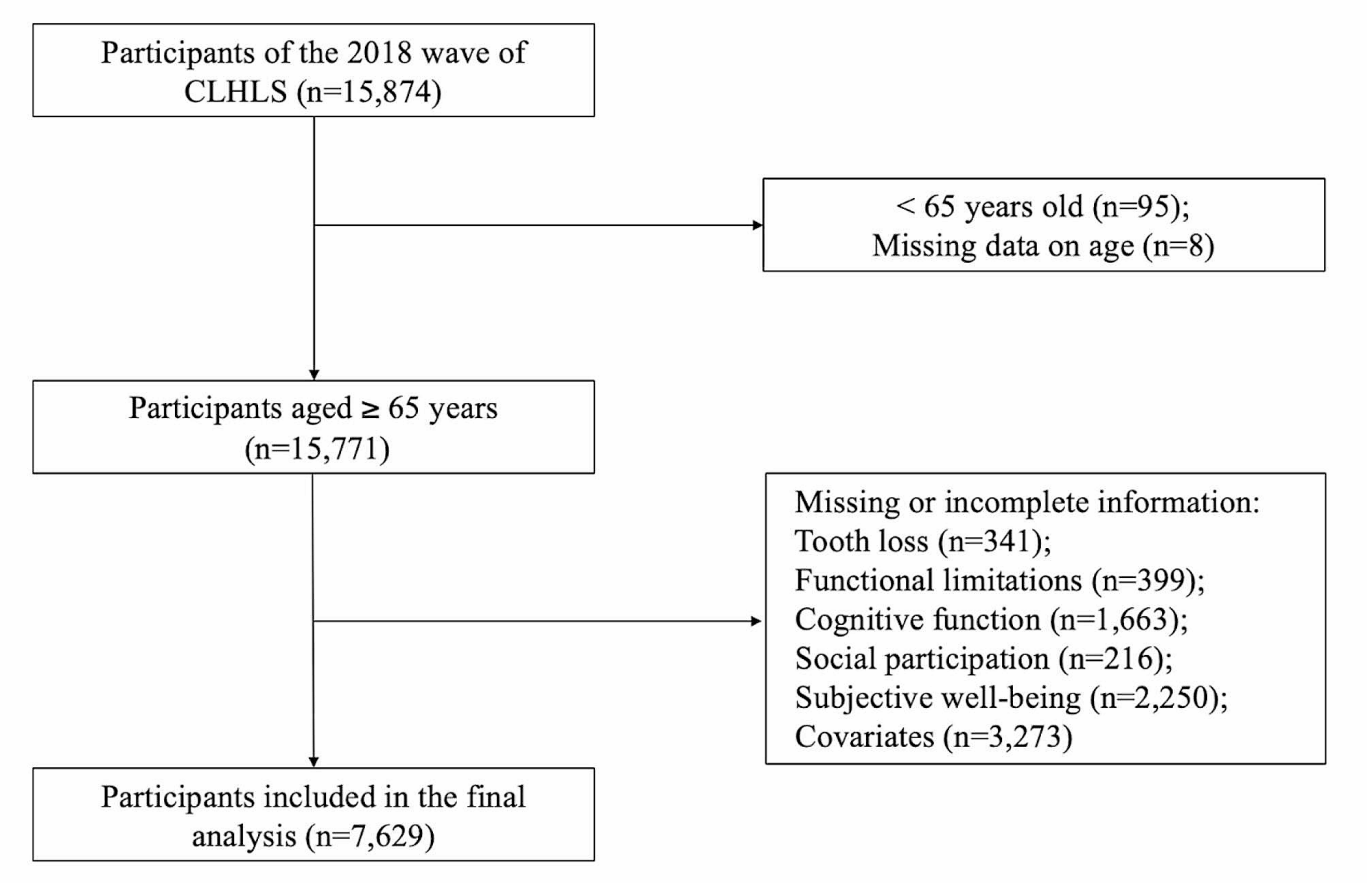
Flowchart of the study population

We used data from the Chinese Longitudinal Healthy Longevity Survey (CLHLS), a nationally representative study covering older adults aged 65 and over in more than 500 localities in 22 provinces and autonomous regions across China. The CLHLS was approved by the ethics committee of Peking University (IRB00001052–13,074), and written informed consent was obtained from all the participants. This study selected the cross-sectional data from 2017/2018 for analysis, comprising a sample of 15,874 participants. We excluded participants with missing data on tooth loss, functional limitations, SWB, social participation, and cognitive function. Data items with missing relevant covariate values were also removed. The final analysis included data from 7,629 participants (Fig. 2).

### Assessments and measurements

#### Tooth loss

Tooth loss was evaluated by self-reporting the presence/absence of natural teeth (ranging from 0 to 32). In our study, we included third molars in the count of present teeth. Because in China, if the third molars are healthy and properly aligned, many adults will retain them instead of extracting them out [28].

#### Functional limitations

Functional limitations included ADL and IADL limitations. ADL limitations were defined as having difficulty with any of six items of the modified Katz index: bathing, dressing, indoor transferring, toileting, continence, and feeding [29]. We defined those who could independently complete these six activities as ADL normal. IADL limitations were defined as having difficulty with any of eight activities in Lawton IADL scale: shopping, visiting neighbors, washing clothes, making food, walking 1 km, crouching and standing (repeated three times), carrying 5 kg weight, and taking public transportation [30]. We defined those who could independently complete these eight activities as IADL normal. The total score served as a numerical indicator, ranging between 0 and 6 for ADL and 0 and 8 for IADL. Higher scores indicated poorer performance of daily activities.

#### Subjective well-being

SWB was defined as a cognitive and emotional evaluation of one’s life, an indicator of life satisfaction, mental health, and happiness [31]. SWB for participants in the CLHLS was based on a previously developed tool using the five-point Likert scale. The tool includes eight items for life satisfaction, positive effects (optimism, happiness, personal control, and conscientiousness), and negative effects (anxiety, loneliness, and uselessness) [32]. Life satisfaction was measured by “How do you rate your life at present?” ranging from 1 (very good) to 5 (very bad). On the emotional side, participants answered the questions, “Do you always look on the bright side of things?” “Are you as happy as when you were younger?” “Can you make your own decisions concerning your personal affairs?” “Do you like to keep your belongings neat and clean?” “Do you often feel fearful or anxious?” “Do you often feel lonely and isolated?” and “Do you feel the older you get, the more useless you are?” The responses to these questions ranged from 1 (always) to 5 (never). We calculated the sum of the three dimensions (life satisfaction, positive emotions, and negative emotions) to represent the overall level of SWB on a scale ranging from 0 to 40 [32]. The higher the score, the stronger the SWB.

#### Social participation

Social participation, also known as social engagement or activity, involves various activities, including facilitator-led group discussions, field trips, and attendance of social groups [33]. In this research, we incorporated three specific social activities into this subdomain: playing cards/mahjong (0 = never; 1 = almost every day/once a week/at least once a month/sometimes), engaging in organized social activities (0 = never; 1 = almost every day/once a week/at least once a month/sometimes), and visiting experiences (0 = 0; 1 = more than 0). The score for social participation ranged from 0 to 3 [34].

#### Cognitive function

Cognitive function was measured by the Chinese version of the Mini-Mental State Examination (MMSE), which considered the cultural and socio-economic conditions of older adults in China, and respondents can easily understand and answer all the questions in the test. It includes 24 items related to orientation, registration, attention, calculation, recall, and language. The total score ranges between 0 and 30. More details can be found in our previous research [22].

#### Covariates

Covariates collected through interviews included sociodemographic, behavioral characteristics, and health-related indicators. Sociodemographic variables included age, sex (male or female), marital status (married or widowed/separated/single), place of residence (urban or town/rural), education level (less or over one year), primary occupation before retirement (non-professional or professional work), and economic status compared with the local residents (poor, ordinary, or wealthy). Behavioral characteristics included recent drinking, smoking, and physical exercises. Health status indicators included body mass index (BMI) and self-reported chronic diseases. BMI was calculated by dividing weight in kg by the square height in m. Self-reported chronic diseases included hypertension, diabetes, heart disease, cerebrovascular disease, pulmonary disease, and cancer. The number of chronic diseases was included in the analysis [22].

### Data analysis

Data were analyzed using IBM SPSS Statistics for Windows, Version 25.0 (IBM Corp., Armonk, NY, USA). The participant demographic characteristics were assessed by independent samples *t*-tests for continuous measures and chi-squared tests for categorical measures. Continuous variables are expressed as means and standard deviations. Categorical variables are expressed as counts and proportions. Pearson correlation analysis evaluated the bivariate associations between the main variables. The PROCESS macro for SPSS (Model 6) was used to test the serial mediation effect. The serial multiple mediation model included seven indirect effect combinations (Indirect 1: X → M1 → Y; Indirect 2: X → M2 → Y; Indirect 3: X → M3 → Y; Indirect 4: X → M1 → M2 → Y; Indirect 5: X → M1 → M3 → Y; Indirect 6: X → M2 → M3 → Y; Indirect 7: X → M1 → M2 → M3→ Y). We treated tooth loss as the independent variable (X), social participation as the first mediator (M1), SWB as the second mediator (M2), cognitive function as the third mediator (M3), and ADL/IADL limitations as the dependent variable (Y). A bootstrap test with 5,000 repeat samplings tested the statistical robustness. A 95% confidence interval (CI) not containing 0 indicated a significant moderating effect [35]. The models were adjusted for age, sex, marital status, place of residence, education level, primary occupation, economic status, drinking, smoking, physical exercises, BMI, and the total number of chronic diseases.

## Results

### Characteristics of the participants

As shown in Table 1, of the 7,629 participants (3,641 males, 3,988 females) included in the analysis,1,187 (15.6%) had ADL limitations, and 4,372 (57.3%) had IADL limitations. Participants with ADL limitations tended to be older, female, with a lower education level, widowed/separated/single, and living in towns or villages. Moreover, people with ADL limitations had lower BMI, less physical exercise, more chronic diseases, more serious tooth loss, a low social participation level, poor SWB, and a low cognitive function score (*p* < 0.001, Table 1). Participants with IADL limitations presented a similar picture (Table S1).

**Table 1:** Characteristics of the participants stratified by functional status

Characteristic	Total	ADL normal	ADL limitations	P-value
	(*n* = 7629)	(*n* = 6442)	(*n* = 1187)	
Age (mean ± SD)	82.30 ± 11.08	80.35 ± 10.28	92.88 ± 9.08	< 0.001
Sex (%)				< 0.001
Male	3641(47.7)	3161(49.1)	480(40.4)	
Female	3988(52.3)	3281(50.9)	707(59.6)	
BMI (mean ± SD)	22.79 ± 4.35	22.92 ± 4.22	22.07 ± 4.96	< 0.001
Education level (%)				< 0.001
< 1 year	3054(40.0)	2373(36.8)	681(57.4)	
≥ 1 year	4575(60.0)	4069(63.2)	506(42.6)	
Marital status (%)				< 0.001
Married	3813(50.0)	3548(55.1)	265(22.3)	
Widowed/Separated/single	3816(50.0)	2894(44.9)	922(77.7)	
Self-reported financial status (%)				0.294
Poverty	671(8.8)	562(8.7)	109(9.2)	
Ordinary	5356(70.2)	4545(70.6)	811(68.3)	
Wealthy	1602(21.0)	1335 (20.7)	267(22.5)	
Working condition (%)				0.218
Non-professional work	6571(86.1)	5562(86.3)	1009(85.0)	
Professional work	1058(13.9)	880(13.7)	178(15.0)	
Residence (%)				< 0.001
Urban	2087(27.4)	1650(25.6)	437(36.8)	
Town/Rural	5542(72.6)	4792(74.4)	750(63.2)	
Smoking (%)	1276(16.7)	1144(17.8)	132(11.1)	< 0.001
Drinking (%)	1239(16.2)	1113(17.3)	126(10.6)	< 0.001
Exercise (%)	2902(38.0)	2642(41.0)	260(21.9)	< 0.001
IADL (mean ± SD)	2.47 ± 2.91	1.73 ± 2.35	6.53 ± 2.24	< 0.001
Number of chronic diseases (mean ± SD)	0.97 ± 1.02	0.94 ± 0.99	1.12 ± 1.16	< 0.001
Number of natural teeth (mean ± SD)	11.24 ± 10.75	12.26 ± 10.85	5.68 ± 8.20	< 0.001
Subjective well-being (mean ± SD)	36.93 ± 4.95	37.17 ± 4.81	35.62 ± 5.50	< 0.001
Social participation (mean ± SD)	0.54 ± 0.79	0.59 ± 0.82	0.23 ± 0.54	< 0.001
Cognitive function (mean ± SD)	26.08 ± 5.34	26.99 ± 4.20	21.18 ± 7.70	< 0.001

*Notes*: SD, standard deviation; BMI, body mass index; ADL, activities of daily living

### Correlation of natural teeth number, social participation, SWB, cognitive function, and functional limitations

As shown in Table 2, correlation analysis indicated that any two variables were interrelated. Specifically, ADL limitations were negatively correlated with the number of natural teeth (*r* = − 0.189, *p* < 0.001), social participation (*r* = − 0.157, *p* < 0.001), SWB (*r* = − 0.149, *p* < 0.001), and cognitive function (*r* = − 0.432, *p* < 0.001). IADL limitations were also negatively correlated with these variables. Cognitive function was positively correlated with the number of natural teeth (*r* = 0.305, *p* < 0.001), social participation (*r* = 0.267, *p* < 0.001), and SWB (*r* = 0.239, *p* < 0.001).

**Table 2:** Correlations for the main variables

Variable	1	2	3	4	5	6
1. Number of natural teeth	-					
2. ADL	–0.189^**^	-				
3. IADL	–0.365^**^	0.548^**^	-			
4. Social participation	0.243^**^	–0.157^**^	–0.330^**^	-		
5. Subjective well-being	0.108^**^	–0.149^**^	–0.220^**^	0.154^**^	-	
6. Cognitive function	0.305^**^	–0.432^**^	–0.550^**^	0.267^**^	0.239^**^	-

Notes**p < 0.001. ADL, activities of daily living; IADL, instrumental activities of daily living;

### Mediating effects of social participation, SWB, and cognitive function in the association between tooth loss and functional limitations

As shown in Table 3, the serial mediation effects model testing indicated that the direct association between tooth loss and ADL limitations was insignificant (β = − 0.0073; 95% CI, − 0.0029 to 0.0015). The model showed that social participation, SWB, and cognitive function mediated the relationship between tooth loss and ADL limitations (β = − 0.0026, − 0.0012, and − 0.0018, respectively). Furthermore, social participation, SWB, and cognitive function were sequentially related, constituting a serial mediation model that indicated significant total (β = − 0.0261; 95% CI, − 0.0049 to − 0.0002) and indirect (β = − 0.0188; 95% CI, − 0.0259 to − 0.0121) effects on the association of tooth loss with ADL limitations.

**Table 3:** Serial mediation effects of social participation, subjective well-being, and cognitive function on the association between tooth loss and ADL limitations

Variable	Path	β	SE	LLCI	ULCI
Total effect	Tooth loss → ADL limitations	–0.0261	0.0012	–0.0049	–0.0002
Direct effect	Tooth loss → ADL limitations	–0.0073	0.0011	–0.0029	0.0015
Total Indirect effect	Tooth loss → ADL limitations	–0.0188	0.0035	–0.0259	–0.0121
Indirect effect	Tooth loss → Social participation → ADL limitations	–0.0026	0.0007	–0.0041	–0.0013
	Tooth loss → SWB → ADL limitations	–0.0012	0.0007	–0.0028	–0.0002
	Tooth loss → Cognitive function → ADL limitations	–0.0118	0.0032	–0.0184	–0.0056
	Tooth loss → Social participation → SWB → ADL limitations	–0.0001	0.0001	–0.0003	–0.0001
	Tooth loss → Social participation → Cognitive function → ADL limitations	–0.0013	0.0003	–0.0020	–0.0007
	Tooth loss → SWB → Cognitive function→ ADL limitations	–0.0015	0.0007	–0.0029	–0.0002
	Tooth loss → Social participation → SWB → Cognitive function→ ADL limitations	–0.0002	0.0001	–0.0003	–0.0001

*Notes*: β, unstandardized coefficients; SE, the standard error of indirect effects estimated; LLCI, lower limit confidence interval; ULCI, upper limit confidence interval; ADL, activities of daily living; SWB, subjective well-being

Unlike ADL limitations, we found a significant direct effect of tooth loss on IADL limitations (β = − 0.0308; 95% CI, − 0.0131 to − 0.0036). The serial mediation effects of social participation, SWB, and cognitive function on the association between tooth loss and IADL limitations remained (Table 4).

**Table 4:** Serial mediation effects of social participation, subjective well-being, and cognitive function on the association between tooth loss and IADL limitations

Variable	Path	β	SE	LLCI	ULCI
Total effect	Tooth loss → IADL limitations	–0.0509	0.0026	–0.0189	–0.0087
Direct effect	Tooth loss → IADL limitations	–0.0308	0.0024	–0.0131	–0.0036
Total Indirect effect	Tooth loss → IADL limitations	–0.0201	0.0031	–0.0265	–0.0141
Indirect effect	Tooth loss → Social participation → IADL limitations	–0.0068	0.0014	–0.0096	–0.0041
	Tooth loss → SWB → IADL limitations	–0.0021	0.0010	–0.0041	–0.0004
	Tooth loss → Cognitive function → IADL limitations	–0.0087	0.0024	–0.0134	–0.0041
	Tooth loss → Social participation → SWB → IADL limitations	–0.0002	0.0001	–0.0004	–0.0001
	Tooth loss → Social participation → Cognitive function → IADL limitations	–0.0010	0.0002	–0.0014	–0.0005
	Tooth loss → SWB → Cognitive function → IADL limitations	–0.0011	0.0005	–0.0021	–0.0002
	Tooth loss → Social participation → SWB → Cognitive function → IADL limitations	–0.0001	0.0001	–0.0002	–0.0001

*Notes*: β, standardized coefficients; SE, the standard error of indirect effects estimated; LLCI, lower limit confidence interval; ULCI, upper limit confidence interval; IADL, instrumental activities of daily living; SWB, subjective well-being

## Discussion

The present study explored the underlying processes affecting the association between tooth loss and functional limitations among older Chinese adults. Social participation, SWB, and cognitive function mediated the relationship between tooth loss and ADL/IADL limitations. Analyses supported the serial mediating role these variables fulfilled. Our findings could deepen the understanding of the role social and psychological factors played in the relationship between oral health and functional limitations and provide a reference for specific interventions to counter functional limitations.

This study found that tooth loss had a significant positive association with functional limitations, consistent with Hypothesis 1 and previous studies that found significant associations between tooth loss and greater odds of mobility limitations or function impairment in older adults [4–6, 19]. This related mechanism can be considered from the causes of tooth loss. In China, among older adults aged 65–74, the prevalence of periodontal disease and permanent tooth decay is 64.6% [36] and 98.0% [37], respectively, which are the main causes of tooth loss [37]. Periodontal diseases are regarded as biofilm-initiated inflammatory conditions, principally gingivitis and periodontitis [38]. Chronic inflammation is considered a biological mechanism for age-related functional decline [7]. Moreover, if periodontitis and caries are not treated properly, they can lead to a decrease in chewing function and poor nutritional status [8, 38], thereby inducing functional disabilities. It is important to note that although our study did not specifically analyze the role of denture wearing in mitigating these functional limitations, the inclusion of denture use as a factor in our analysis was considered. Preliminary findings suggested the impact of dentures on the relationship between tooth loss and functional limitations was minimal, leading to their exclusion from the final analysis. However, we acknowledge the potential importance of dentures in preserving chewing function and nutritional status, thereby potentially reducing the impact of tooth loss on functional limitations. Future research should delve deeper into the role of dentures, including types and quality of dentures, in this complex relationship to provide a more nuanced understanding of how dentures may serve as a compensatory mechanism for tooth loss. In addition, social problems caused by tooth loss are also considered as an important reason for functional limitations [19], but confirmatory empirical research is lacking.

Consistent with Hypothesis 2, we found that social participation significantly mediated the relationship between tooth loss and functional limitations. Specifically, those with significant tooth loss were observed to have a reduced social participation level, which in turn was associated with increased functional limitations. Another study utilizing data from CLHLS similarly indicated that social participation had a significant relationship with the number of remaining teeth [39]. However, in this study’s hypothesis, social participation was considered the cause, and tooth loss was viewed as the outcome. This suggested the possibility of a reciprocal relationship between social participation and tooth loss. In other words, tooth loss might affect social participation, and decreased social participation could further impact an individual’s oral health. Concurrently, active social participation might contribute to maintaining or improving oral health, thereby reducing the risk of tooth loss. Nevertheless, there was some uncertainty in this assumption since the two studies differed in their definitions and measurements of social participation. To more comprehensively understand this complex relationship, further research is necessary to directly investigate this bidirectional interaction. A study in China revealed that eating and communication difficulties played a mediating role in the association between tooth loss and functional limitations [19]. The inevitable outcome of the impact tooth loss has on eating and communication is that older adults reduce their social participation [9]. Therefore, promoting social participation of older adults with few or no teeth might help prevent the occurrence of functional limitations.

We also found that SWB mediated the relationship between tooth loss and functional limitations, in line with Hypothesis 3. Our findings align with previous research demonstrating the connection between tooth loss and SWB [14] and SWB and functional limitations [40]; however, those studies did not consider the mediating role of SWB. The disablement process model emphasizes that psychological factors such as SWB might mediate and affect the path from disease accumulation to functional limitations [20]. Notably, most correlational studies indicated that functional health was an important prerequisite for SWB [41–42]. However, Martin et al. emphasized the reciprocal relationship between SWB and physical function. SWB could affect physical function because positive emotions motivate individuals to exhibit healthy behaviors [40]. Although the current conclusion is consistent with the disablement process model, the longitudinal mediating role of SWB in the relationship between tooth loss and functional limitation should be further investigated.

Supporting Hypothesis 4, this study showed a mediating role for cognitive function in the association between tooth loss and functional limitation. A longitudinal study in England reported that total tooth loss was independently associated with physical and cognitive decline in older adults [43]. Our research expands the conclusions of previous research by introducing cognitive function as a mediating factor. Tooth loss could lead to declined cognitive function through several mechanisms, including reduced chewing ability that might affect cerebral blood flow or systemic inflammation [44]. In turn, this impaired cognitive function could lead to difficulties in executing daily tasks, thereby substantiating the functional limitations observed.

In line with Hypothesis 5, this study found a serial mediating effect of social participation, SWB, and cognitive function in the path between tooth loss and functional limitations. Based on positive gerontology [45], individuals with tooth loss usually experience poor social participation, unconducive to developing a sense of community belonging and environmental control. These, in turn, are unconducive to the mental health and happiness of older adults. Active social participation could provide emotional support and cognitive stimulation, which could serve as a buffer against the functional limitations one might experience due to tooth loss [45]. Evidently, functional health needs more than just dental health care measures. Our research calls on public health policymakers and professionals to adopt a more integrated approach that considers social and psychological ways to improve older people’s health and well-being.

We acknowledge several limitations to this study. First, our findings were based on cross-sectional analyses, so the results cannot establish a causal relationship between tooth loss and functional limitations. The possibility of reverse causality between variables cannot be ruled out based on observational analysis. This limitation is particularly pertinent to our exploration of the mediating roles of social participation, SWB, and cognitive function. While these hypotheses are grounded in theoretical frameworks suggesting plausible pathways, the cross-sectional nature of our study limits our capacity to confirm causality or the directionality of these relationships. Future longitudinal studies are needed to more definitively test these mediating pathways. Second, tooth loss was based on self-report rather than a professional oral examination. Although self-reported tooth loss is a common method in large-scale investigations and has been proven to be effective and reliable [46], we acknowledge the challenges older adults may face in accurately counting their teeth. This could lead to either overestimation or underestimation of tooth loss. Therefore, our results should be interpreted with caution. Furthermore, detailed information about the types of tooth loss was unavailable. Future research could examine how various tooth loss types and extents impact the mediation pathways identified. For example, do the effects differ if the tooth loss is in a visible or non-visible area or if the individual uses dental prosthetics? Third, although we controlled for many potential covariates, oral health behavior factors were not included, and these might affect the observed association. Fourth, besides the strong correlation observed between ADL and IADL, the correlations among other major variables appear to be low. This suggests that certain crucial factors, potentially including biological and pathological ones, might not have been adequately accounted for in the study examining the link between tooth loss and functional status. In future research, it will be important to consider these factors to more accurately explore and elucidate their relationships.

In conclusion, the present study revealed that social participation, SWB, and cognitive function serially mediated the association between tooth loss and functional limitations. Our findings provide a new perspective for exploring the pathways linking oral health and functional limitations and underscore the necessity of considering psychological and social factors as integrated healthcare approaches to preserve the functional health of older adults.

### Electronic supplementary material

Below is the link to the electronic supplementary material.

Supplementary Material 1

## Data Availability

The original contributions presented in this study are included in the article material, further inquiries can be directed to the corresponding authors.

## Abbreviations

ADL: Activities of daily living
IADL: Instrumental activities of daily living
SWB: Subjective well-being
CLHLS: Chinese Longitudinal Healthy Longevity Survey
MMSE: Mini-Mental State Examination
BMI: body mass index
