# Supplementary material for: Social participation, subjective well-being, and cognitive function as serial mediators between tooth loss and functional limitations in older Chinese adults
Source: BMC Public Health. 2024 Mar 14;24:803. doi: 10.1186/s12889-024-18255-w (PMC10938731; doi:10.1186/s12889-024-18255-w)
Supplement: Supplementary file 1 — Supplementary Material 1 [file 12889_2024_18255_MOESM1_ESM.docx]

Supplementary Material

# Supplementary Tables

- Table S1 Characteristics of the participants stratified by functional status.

| **Table S1. Characteristics of the participants stratified by functional status.** | | | | |
| --- | --- | --- | --- | --- |
| **Characteristic** | **Total** | **IADL**  **normal** | **IADL limitations** | ***P*-value** |
|  | **(n=7629)** | **(n=3257)** | **(n=4372)** |  |
| Age (mean ± SD) | 82.30 ± 11.08 | 75.24 ± 7.91 | 87.56 ± 10.13 | <0.001 |
| Sex (%) |  |  |  | <0.001 |
| Male | 3641(47.7) | 1873(57.5) | 1768(40.4) |  |
| Female | 3988(52.3) | 1384(42.5) | 2604(59.6) |  |
| BMI (mean ± SD) | 22.79 ± 4.35 | 23.39 ± 4.07 | 22.34 ± 4.50 | <0.001 |
| Education level (%) |  |  |  | <0.001 |
| < 1 year | 3054(40.0) | 741(22.8) | 2313(52.9) |  |
| ≥ 1 year | 4575(60.0) | 2516(77.2) | 2059(47.1) |  |
| Marital status (%) |  |  |  | <0.001 |
| Married | 3813(50.0) | 2282(70.1) | 1531(35.0) |  |
| Widowed/separated/single | 3816(50.0) | 975(29.9) | 2841(65.0) |  |
| Self-reported financial status (%) |  |  |  | <0.001 |
| Poverty | 671(8.8) | 247(7.6) | 424(9.7) |  |
| Ordinary | 5356(70.2) | 2274(69.8) | 3082(70.5) |  |
| Wealthy | 1602(21.0) | 736(22.6) | 866(19.8) |  |
| Working condition (%) |  |  |  | <0.001 |
| Non-professional work | 6571(86.1) | 2697(82.8) | 3874(88.6) |  |
| Professional work | 1058(13.9) | 560(17.2) | 498(11.4) |  |
| Residence (%) |  |  |  | 0.533 |
| Urban | 2087(27.4) | 903(27.7) | 1184(27.1) |  |
| Town/Rural | 5542(72.6) | 2354(72.3) | 3188(72.9) |  |
| Smoking (%) | 1276(16.7) | 736(22.6) | 540(12.4) | <0.001 |
| Drinking (%) | 1239(16.2) | 726(22.3) | 513(11.7) | <0.001 |
| Exercise (%) | 2902(38.0) | 1619(49.7) | 1283(29.3) | <0.001 |
| ADL (mean ± SD) | 0.35 ± 1.04 | 0.02 ± 0.21 | 0.60 ± 1.31 | <0.001 |
| Number of chronic diseases (mean ± SD) | 0.97 ± 1.02 | 0.89 ± 0.96 | 1.02 ± 1.06 | <0.001 |
| Number of natural teeth (mean ± SD) | 11.24 ± 10.75 | 15.35 ± 10.93 | 8.17 ± 9.52 | <0.001 |
| Subjective well-being (mean ± SD) | 36.93 ± 4.95 | 38.11 ± 4.57 | 36.05 ± 5.04 | <0.001 |
| Social participation (mean ± SD) | 0.54 ± 0.79 | 0.81 ± 0.90 | 0.33 ± 0.63 | <0.001 |
| Cognitive function (mean ± SD) | 26.08 ± 5.34 | 28.46 ± 2.22 | 24.31 ± 6.22 | <0.001 |
| Notes: SD, standard deviation; BMI, body mass index; IADL, instrumental activities of daily living; | | | | |
